# Intramuscular Versus Intravenous Treatment of Status Epilepticus: A Systematic Review

**DOI:** 10.7759/cureus.38212

**Published:** 2023-04-27

**Authors:** Sarah S Aldharman, Faisal T Alayed, Fay A Almutairi, Badr S Aljohani, Kadi A Alhumaidi, Abdulaziz S Alayyaf, Rayan M Alismail, Fahad H Binshalhoub, Shahad J Alsahil, Saud A Alnaaim

**Affiliations:** 1 College of Medicine, King Saud Bin Abdulaziz University for Health Sciences, Riyadh, SAU; 2 College of Medicine, Al-Imam Mohammad Ibn Saud Islamic University, Riyadh, SAU; 3 College of Medicine, Majmaah University, Riyadh, SAU; 4 College of Medicine, Al-Rayan Medical Colleges, Madina, SAU; 5 College of Medicine, Qassim University, Al-Qassim, SAU; 6 College of Medicine, Prince Sattam Bin Abdulaziz University, Al-Kharj, SAU; 7 College of Medicine, Al-Imam Mohammed Ibn Saud Islamic University, Riyadh, SAU; 8 College of Medicine, King Saud University, Riyadh, SAU; 9 Department of Clinical Neurosciences, King Faisal University, Al-Ahsa, SAU

**Keywords:** review, treatment, intravenous, intramuscular, status epilepticus

## Abstract

Status epilepticus is a neurological emergency associated with high morbidity and mortality with fatal outcomes if not treated well. The goal of this study was to compare the intramuscular and intravenous treatment of individuals with status epilepticus. A search was performed on Scopus, PubMed, Embase, and Web of Science databases for articles published in the English language in peer-reviewed publications up to March 1, 2023. Studies were included if the treatment of status epileptics was compared either directly or indirectly between intramuscular and intravenous methods. In addition, relevant papers were manually screened for in the reference lists of the included studies. Non-duplicate articles were identified. Finally, five articles were included in the analysis, of which four were randomized controlled trials and one was a retrospective cohort study. The intramuscular midazolam group’s time until the first seizure stopped was significantly shorter than the intravenous diazepam group’s time (7.8 versus 11.2 minutes, respectively; p = 0.047). Moreover, the percentage of patients admitted was significantly lower in the intramuscular group than in the intravenous group (p = 0.01), but the lengths of stay in the intensive care unit and the hospital did not differ significantly between the groups. Regarding seizure recurrence, the intramuscular group had fewer incidences of recurrent seizures. Finally, there were no appreciable differences in safety outcomes between the two treatment arms. During the analysis, different outcomes reported after the use of intramuscular and intravenous treatments in managing patients with status epilepticus were categorized. This categorization led to a clear view of the efficacy and safety of intramuscular versus intravenous treatments in managing status epilepticus patients. The information at hand indicates that intramuscular therapy is just as successful as intravenous therapy in treating people with status epilepticus. The availability, adverse effect profile, logistics of administration, cost, and whether it is included in hospital formularies are some of the factors to be taken into consideration when choosing the drug administration technique.

## Introduction and background

Status epilepticus was previously described as a seizure lasting for 30 minutes or more, or a series of seizures in which the patient does not restore normal mental status between convulsions. The 2012 Neurocritical Care Society guidelines updated the definition to a seizure with five minutes or more of continuous clinical and/or electrographic seizure activity or repeated seizure activity without recovery between seizures [1]. It is a serious condition that needs to be treated urgently to prevent further damage to the patient’s life [1]. According to Rossetti et al. [2] and Schubert-Bast et al. [3], there are 9.9 to 41 cases of status epilepticus for every 100,000 people, with a mortality risk of up to 22%. One research estimated the direct cost in the United States to be $4 billion annually, making it a condition that is expensive for healthcare systems [4].

The initial acute therapy for status epilepticus, benzodiazepines, is the same regardless of the etiology or age of the patient, despite the fact that it has a variety of different triggers [5]. Patients with status epilepticus typically receive intravenous diazepam or lorazepam as the first-line treatment [6]. However, it can be difficult and time-consuming to set up an intravenous line in patients during a convulsion, particularly before arriving at the hospital. Although intravenous administration of antiepileptic medications is the recommended method of therapy in status epilepticus, intramuscular administration may be an effective alternative when venous access is problematic [7].

Few randomized clinical trials (RCTs) have explicitly compared intravenous and intramuscular management of status epilepticus [8-10]. Intramuscular treatment can be rapidly administered and has shown a more rapid cessation of seizures in status epilepticus in comparison to the intravenous route [8-10]. There is presently no systematic review that offers a thorough synthesis of the available data. To resolve the discrepancies and offer a more thorough and reliable evaluation of the effectiveness and safety of intramuscular versus intravenous administration of medications in managing status epilepticus, a systematic review on this subject is required.

This review will aid in locating any potential knowledge gaps, provide a more thorough understanding of the distinctions between the two routes of administration, and aid in the creation of clinical guidelines for the management of status epilepticus that are based on the best available evidence to ensure the optimal outcomes for patients. With regard to treating patients with status epilepticus, this study aimed to systematically review all relevant literature that compared intramuscular and intravenous methods in status epilepticus treatment.

## Review

Methodology

The Preferred Reporting Items for Systematic Reviews and Meta-analysis (PRISMA) protocol was followed in this systematic review [11]. To find pertinent studies, check study eligibility, and rate the quality, systematic methods were employed.

Eligibility Criteria

Only English-language articles published as of March 1, 2023, qualified. There was no consideration for gray literature because only published studies were taken into account in this study.

Inclusion Criteria

Articles accessible in the English language and published in peer-reviewed publications were considered for inclusion. Studies were included if the treatment of status epilepticus was compared either directly or indirectly between intramuscular and intravenous methods, and if the articles reported on the effectiveness and safety of the two interventions.

Exclusion Criteria

Systematic reviews, meta-analyses, papers published in journals other than peer-reviewed ones, conference proceedings, author letters, and comments on already published articles were excluded from this review. In addition, studies that did not directly or indirectly compare intramuscular and intravenous administration methods for treating individuals with status epilepticus were excluded.

Literature Search and Screening

Utilizing the OVID interface and the search terms “intramuscular” and “Intravenous,” as well as combinations of search terms describing status epilepticus, a thorough literature search was conducted using the PubMed, Web of Science, Embase, and Scopus databases from the time of their creation through March 1, 2023. Additionally, pertinent articles were manually searched for in the reference lists of the included studies. Two evaluators used the Endnote X9 program to perform a duplicate title, abstract, and full-text screening. Studies were disqualified at the full-text screening step if some of the pre-established eligibility requirements were not met. Any disagreements among the reviewers were resolved through discussion until an agreement was reached.

Data Extraction

Data were extracted in duplicate by at least two reviewers using a standardized extraction form that encompassed the study characteristics (lead author and publication year, study design, and sample size), patient characteristics (age), primary outcomes, and the findings of the study.

Risk of Bias (Quality) Assessment

The Cochrane Collaboration’s risk-of-bias instrument [12] was used to assess the quality of the four included RCTs. The seven components of this tool are as follows: random sequence generation (selection bias), allocation concealment (selection bias), participant and staff blinding (performance bias), outcome assessment blinding (detection bias), incomplete outcome data (attrition bias), selective reporting (reporting bias), and other bias. Every one of the aforementioned components could be ranked in one of three ways, namely, high, unclear, and low risk. The quality of the retrospective cohort study was evaluated using the Newcastle-Ottawa Scale (NOS) [13]. Three elements make up the scale for reporting participant selection, comparability, and result evaluation. The overall grade score for the NOS scale is 8.

Data Synthesis

After gathering the data, we found that the heterogeneity in the reported results and the assessment methods used made it impossible to synthesize evidence quantitatively. Instead, a thematic narrative form of our findings is presented. Additionally, a pooled analysis was conducted for studies that reported outcome data.

Results

Study Selection

**Figure 1:**
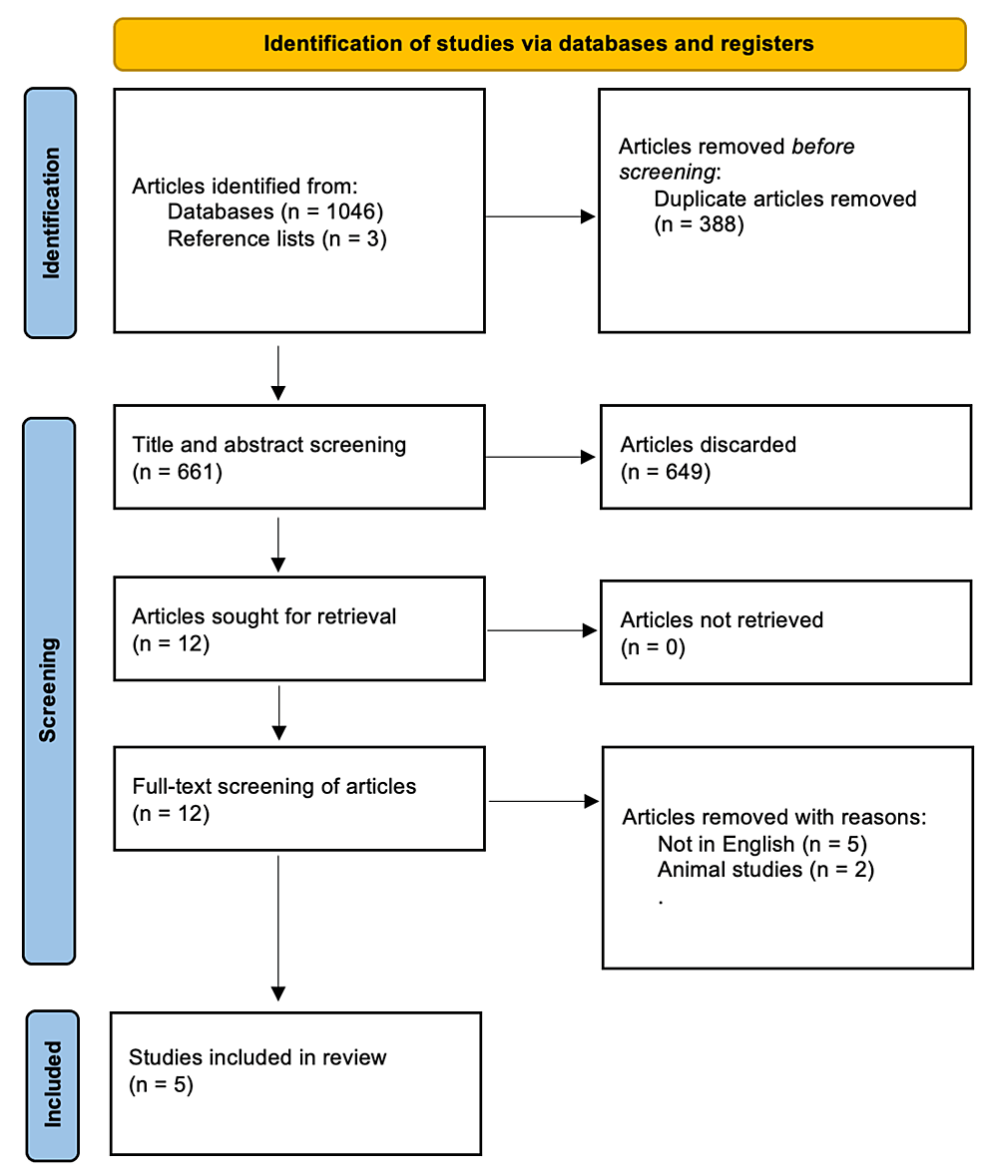
Preferred Reporting Items for Systematic Reviews and Meta-Analyses (PRISMA) flow diagram.

In total, 1,049 entries were found in the literature search, including 613 articles from Embase, 131 articles from Web of Science, 184 articles from Scopus, and 118 articles from PubMed. Following a review of the identified studies’ reference lists, three papers were found. After removing duplicate records, a total of 661 records were left for the title and abstract screening. During the title and abstract screening, a total of 649 papers were disqualified. They either did not report on the use of intramuscular versus intravenous administration in the treatment of status epilepticus or they followed a research methodology mentioned in the exclusion criteria. The remaining 12 studies were read in full, and only five studies met the inclusion criteria. Figure 1 shows a flow diagram of the literature search and screening at different phases during the review process.

Characteristics of the Included Studies

In total, five articles were included in this review. Three of them were RCTs, one was a clinical trial, and one was a cohort study. The study sample varied from 24 to 893. The included studies were published between 1997 and 2015. Table 1 summarizes the characteristics of the included studies. 

**Table 1:** Characteristics of the included studies. IM: intramuscular; IV: intravenous; RCT: randomized controlled trial

Author and publication year	Study design	Sample size	Age of the patients	Primary outcome	Type of comparison	Study findings
Chamberlain et al., 1997 [8]	RCT	24	˂18 years	Time to cessation of seizures	Direct	When compared to IV diazepam, IM midazolam caused seizures to end more quickly (7.8 minutes as opposed to 11.2 minutes; p = 0.047)
Silbergleit et al., 2012 [9]	RCT	893	0–102 years	Cessation of seizures prior to the emergency department	Direct	329/448 participants (73.4%) in the IM midazolam group and 282/445 (63.4%) in the IV lorazepam group experienced no seizures
Welch et al., 2015 [10]	RCT	120	˂18 years	Seizure cessation prior to the emergency department	Direct	41 (68.3%) and 43 (71.7%) of the subjects in the IM and IV groups, respectively, achieved the main outcome
Chen et al., 2009 [14]	Clinical trial	48	2–79 years	Cessation of seizures	Indirect	Within one hour, IV halted 87.5% of patients’ seizures, and after one hour, patients’ mental states returned to normal
Uysal et al., 2015 [15]	cohort study	43	Mean 56.7 ± 14.7 years	Response to treatment and in-hospital death	Direct	74% of patients who received IV anesthetic medication had resolved status epilepticus

Quality Assessment

**Figure 2:**
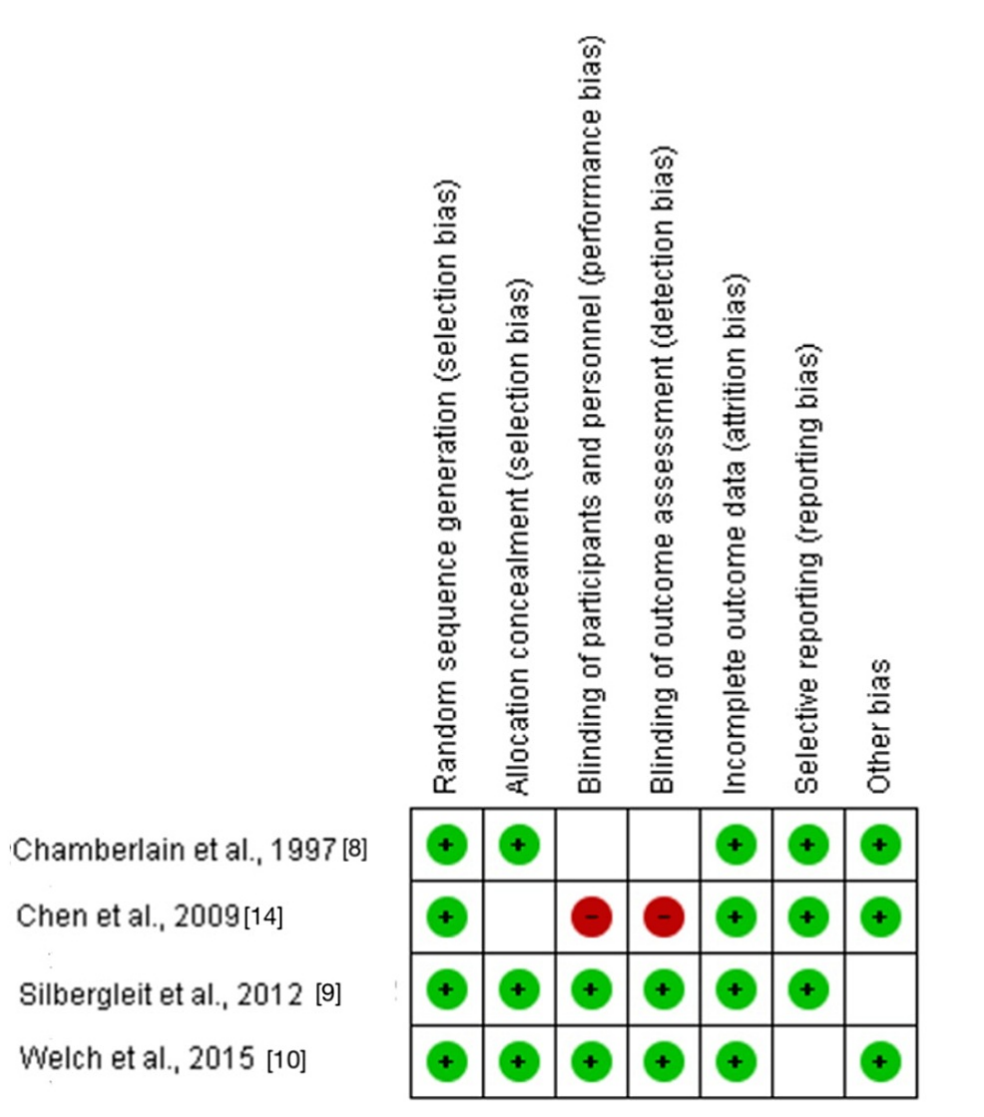
Risk of bias summary for the included trials.

Figure 2 displays the quality assessment findings. Random sequence generation (criterion 1) received a low-risk rating for four (100%) of the studies. Allocation concealment (criterion 2) received a low-risk rating for three (75%) studies and an unclear-risk rating for one (25%) study. In two (50%) studies, criterion 3 was rated as low risk, while one (25%) study had a high-risk rating. Two (50%) studies received low-risk scores for criterion 4, while one (25%) received unclear-risk scores and another a high-risk (25%) score. In four (100%) trials, criterion 5 was rated as low risk. In three (75%) trials, criteria 6 and 7 received a low-risk rating, while in one study (25%) they received an unclear-risk rating. Table 2 shows the quality assessment for the included cohort study.

**Table 2:** Quality assessment for the included cohort study. NOS: Newcastle-Ottawa Scale

Study	Selection	Comparability	Outcome	Total NOS score
Uysal et al., 2015 [15]	3	2	3	8

Cessation of Seizures

Cessation of seizures refers stoppage of the seizure episode. Children with status epilepticus who presented to the emergency room were randomly allocated to receive intramuscular midazolam 0.2 mg/kg or intravenous diazepam 0.3 mg/kg in a prospective, randomized trial by Chamberlain and colleagues [8]. Patients who were receiving anticonvulsants for the present seizure episode or who had an intravenous line in place were disqualified. Patients who arrived at the research location had ongoing seizures that lasted between 14 and 213 minutes. Time until the first seizure stopped was the main outcome, and the intramuscular midazolam group’s time to that point was significantly shorter than the intravenous diazepam group’s time (7.8 versus 11.2 minutes, respectively; p = 0.047). After the initial seizure cessation, four patients in each group experienced recurrent seizures that required the use of additional anticonvulsants.

Patients aged 0-102 years were randomly assigned to either one of the two treatment arms in the largest randomized, double-blinded trial in this analysis by Silbergleit and colleagues [9]: 10 mg of intramuscular midazolam or 4 mg of intravenous lorazepam. Patients with major trauma as the acute precipitant of the seizures, hypoglycemia, cardiac arrest, or a heart rate of fewer than 40 beats per minute (as these conditions require alternative treatments), as well as those who were known to be pregnant, in prison, receiving treatment for another study, or who had a known allergy to midazolam or lorazepam, were excluded from the study. Both groups shared comparable baseline characteristics. Regardless of whether rescue therapy was used or not, subjects randomly assigned to the intramuscular group were less likely to be experiencing seizures when they arrived at the emergency room than those randomly assigned to the intravenous group (proportion of subjects without seizures = 83.9% vs. 76.2%; difference = 7.7 percentage points; 95% CI = 2.5 to 12.9). The percentage of patients admitted was significantly lower (and the percentage of patients discharged from the emergency room was significantly higher) in the intramuscular group than in the intravenous group (p = 0.01), but the lengths of stay in the intensive care unit (ICU) and in the hospital did not differ significantly between the groups. According to Silbergleit et al. [9], the median duration to active treatment among patients whose seizures stopped before arriving at the emergency room was 1.2 minutes for the intramuscular midazolam group and 4.8 minutes for the intravenous lorazepam group.

Welch and colleagues [10] enrolled patients under the age of 18 who had status epilepticus and presented to the emergency room for their double-blinded, multicenter randomized controlled study. In total, 60 patients received intravenous lorazepam, and 60 patients received intramuscular midazolam. Patients who needed alternative treatment for their seizures (due to major trauma, hypoglycemia, cardiac arrest, or heart rates below 40 beats per minute), as well as those who had a known allergy to the study drug, were excluded from the study. The groups’ initial traits were comparable. In comparison to 43 of 60 (71.6%) patients who received intravenous lorazepam treatment, 41 of 60 (68.3%) patients who received intramuscular midazolam treatment met the main outcome of seizure cessation before arrival at the emergency room (risk difference (RD) = -3.3%; 99% CI = -24.9% to 18.2%). Similar findings were found for patient characteristics, seizure etiology and characteristics, in-hospital events, and the main outcome when stratified by the specified age subgroups. Although the point estimates for treatment differences favored the intravenous group for the two higher age strata (intramuscular therapy was preferred for those under the age of six), caution must be exercised when interpreting these subgroup findings due to the extremely small number of patients in each group [10].

Chen and colleagues [14] examined the short-term safety and effectiveness of treating patients with intravenous sodium valproate for diazepam-refractory convulsive status epilepticus in their clinical research. They prospectively recorded 48 patients with refractory convulsive status epilepticus who received intravenous sodium valproate (30 mg/kg, 6 mg/kg per hour) at West China Hospital following the failure of a loading dose of intravenous diazepam and intramuscular phenobarbitone. Anesthesia was not required because sodium valproate intravenously controlled convulsive status epilepticus in 42 (87.5%) cases (22 patients within 20 minutes, and 20 patients within 60 minutes). After the seizure had stopped, all 42 patients returned to their normal mental state within an hour, and none of the patients had another convulsive seizure over the course of the following 12 hours. However, two patients exhibited decreased frequency and duration of seizures throughout their hospital stay.

Uysal and colleagues [15] examined the medical records of 43 status epilepticus patients admitted between January 1, 2009, and June 30, 2014, at the University of Kansas Medical Center, Kansas City. Patients who fulfilled the clinical criteria for non-conclusive status epilepticus were included. Patients were disqualified if they presented with simple partial status epilepticus, inadequate clinical documentation of their results, a Creutzfeldt-Jacob disease diagnosis, or anoxic brain injury. Overall, 74% of patients who received intravenous anesthetic medications experienced non-conclusive status epilepticus improvement. The duration of status epilepticus did not differ significantly between the treatment groups (intravenous anesthetic medication users vs. non-users group). Furthermore, there were 13 in-hospital deaths (10 in intravenous anesthetic medication users vs. three in the non-users group). One in-hospital death appeared to be a direct consequence of intravenous anesthetic medication use. There was no discernible difference between therapy groups in the duration of non-conclusive status epilepticus.

Recurrence of Seizures

There was no difference between the intramuscular and intravenous groups in terms of recurrent seizures following medication in the study by Chamberlain and colleagues [8]. In their trial, in each treatment arm, there were four individuals who experienced recurrent seizures. Similar results were reported in the study by Silbergleit and colleagues [9]. According to Silbergleit et al., the incidence of recurrent seizures was comparable in the intramuscular and intravenous study groups (11.4% and 10.6%, respectively) [9].

In terms of seizure recurrence, the study by Wells and colleagues [10] found that in comparison to the intravenous group, the intramuscular group was linked with fewer recurrent seizures.

Safety Outcomes

There were no documented complications in the trial by Chamberlain et al., and none of the patients displayed any clinical signs of respiratory depression. Thus, intramuscular and intravenous administration were comparable in terms of safety [8]. According to consistent results from the study by Silbergleit et al. [9], there were no appreciable differences in safety outcomes between the two treatment arms. Although four patients in the study by Chen et al. were unresponsive to intravenous sodium valproate and died within 60 minutes of multiple organ failure, they found no evidence of systemic or local side effects associated with the medication [14].

According to Welch et al. [10], fewer patients in the intramuscular group (60 in the intravenous group vs. 60 in the intramuscular) experienced respiratory failure and required intubation or ICU admission than those receiving intravenous medication. About eight (13%) patients who received intramuscular midazolam required ICU admission, while 13 (22%) of those who received intravenous lorazepam were admitted to the ICU. Moreover, five (8%) patients in the intramuscular midazolam group required intubation, whereas nine (15%) patients in the intravenous lorazepam group required intubation. In a retrospective cohort analysis conducted by Uysal and colleagues [15], there were a total of 13 in-hospital deaths, with 10 occurring in patients who used intravenous anesthetic drugs versus three in the group who did not (p > 0.05). Only one hospital fatality appeared to have been caused directly by intravenous anesthetic use.

Discussion

The goal of this study was to compare intramuscular and intravenous management of individuals with status epilepticus. This is the first time intramuscular and intravenous management of status epilepticus has been compared in a systematic review. This review included three RCTs, one clinical trial, and one cohort study. Results from this review suggest that intravenously treated patients had a non-significantly higher chance of seizure cessation than those treated intramuscularly [10,14,15]. However, the biggest trial included in this study reported that more patients in the intramuscular group than in the intravenous group experienced seizure cessation (73.4% vs. 63.4%, respectively; p = 0.001) [9].

Regarding the time to seizure cessation, evidence from this study shows that intramuscular management is associated with less time to seizure cessation compared to intravenous management (7.8 vs. 11.2 minutes; p = 0.047). However, this outcome was reported by only one trial which included only 24 patients [8]. According to Silbergleit et al. [9], the median duration to active treatment among patients whose seizures stopped before arriving at the emergency room was 1.2 minutes for the intramuscular midazolam group and 4.8 minutes for the intravenous lorazepam group.

Understanding the safety profile of each drug depends on the reporting of safety results in drug comparisons [16]. It offers details on the advantages and disadvantages of each medicine, assisting patients, physicians, and other healthcare professionals in choosing the right medication. Evidence from this research suggests that intramuscular and intravenous treatment are equally safe, as the frequency of complications and unfavorable events in the two study groups was generally comparable [8,9,14,15]. This finding is also similar to another study that found that there were no differences in complications when applying midazolam either intramuscularly or intravenously [17]. However, one of the studies in this review reported contradicting evidence [10]. According to Welch et al., compared to the intravenous group, the intramuscular group experienced fewer complications, such as respiratory failure and hypotension. However, it is not clear if the difference was significant or not [10].

The small number of studies, high risk of bias in some of the studies, and heterogeneity in terms of the participants examined and definitions of status epilepticus cessation are some possible limitations of this systematic review. Only five studies were eligible for inclusion in this review, and each trial had anywhere from 24 to 893 participants. The only trial that reported a power estimate for their sample size was the largest study conducted by Silbergleit et al. [9]. Despite the fact that the majority of the studies included in this systematic review were RCTs, some of the included RCTs were rated as having a high risk or unclear risk of bias in at least one domain. Lack of blinding of the research staff or outcome assessors was the most frequent cause of unclear or high risk of bias. Participant ages, meanings of status epilepticus, and definitions of status epilepticus cessation varied across studies. Therefore, additional research, particularly RCTs, is required before drawing firm conclusions about the effectiveness of the two therapeutic choices. Moreover, it is recommended that future studies investigate these two treatment methods (intravenous vs. intramuscular) in terms of pre-hospital and in-hospital management of status epilepticus.

## Conclusions

For the treatment of status epilepticus, intramuscular therapy appears to be equally effective as intravenous therapy. Depending on the definition used, the intramuscular treatment efficacy rates for status epilepticus cessation varied from 68.3% to 73.4%, while the intravenous treatment efficacy rates ranged from 63.4% to 87.5%. The included trials revealed no statistically significant differences among agents for secondary outcomes, including adverse effects, despite possibly being underpowered to allow for definitive statements. Compared to intravenous therapy, intramuscular therapy leads to fewer complications, such as intubation and ICU admission. On a patient-specific basis, additional variables, such as drug interactions, comorbidities, administration logistics, availability, and expense, may be taken into account to choose the drug of first preference.
